# Longitudinal association between egg consumption and the risk of cardiovascular disease: interaction with type 2 diabetes mellitus

**DOI:** 10.1038/s41387-018-0033-1

**Published:** 2018-04-25

**Authors:** Jiyoung Jang, Min-Jeong Shin, Oh Yoen Kim, Kyong Park

**Affiliations:** 1 Department of Food and Nutrition, Yeungnam University, Gyeongsan, Republic of Korea; 2 Department of Public Health Sciences, BK21PLUS Program in Embodiment: Health-Society Interaction, Graduate School, Korea University, Seoul, Republic of Korea; 3 Department of Food Science and Nutrition, Dong-A University, Busan, Republic of Korea

## Abstract

**Background/objectives:**

It remains unclear if high egg consumption has beneficial or adverse effects on cardiometabolic health. The present study prospectively evaluated the longitudinal association between egg-consumption levels and incident cardiovascular disease (CVD) among Korean adults.

**Subjects/methods:**

We conducted a prospective cohort study of 9248 Korean adults aged 40–69 years without CVD or cancer at the baseline from the Korean Genome and Epidemiology Study, Ansung–Ansan cohort, South Korea. The egg intake of the participants was estimated using a validated semiquantitative food frequency questionnaire at the baseline and the second follow-up examination and categorized into quartiles. CVD cases were identified using biennial questionnaires and confirmed through repeated in-depth personal interviews. Hazard ratios (HR) and 95% confidence interval (CI) were analyzed using Cox proportional hazard regression.

**Results:**

During the average follow-up of 7.3 years, 570 cases of CVD were newly diagnosed. After adjusting for multiple confounding variables, egg-intake levels were not associated with CVD incidence (HR: 1.14, 95% CI: 0.87–1.49, *P* for trend: 0.7). However, the association was modified by type 2 diabetes mellitus (T2DM) status. Egg consumption was significantly associated with an increased risk for incident CVD among participants with T2DM; individuals with the highest egg intake (4.2 ± 0.04 eggs/week) had a 2.8 times higher incidence of CVD (HR: 2.81, 95% CI: 1.25–6.30, *P* for trend: 0.02) than those with the lowest egg intake (0.1 ± 0.02 eggs/week). However, no association was observed among individuals without T2DM (HR: 1.03, 95% CI: 0.77–1.38, *P* for trend: 0.8).

**Conclusions:**

Higher egg consumption may increase the risk for CVD in Korean patients with T2DM. Our findings provide a basis for the development of an optimal dietary cholesterol intake guideline for the Korean population.

## Introduction

Although eggs are an affordable and healthy source of high-quality protein and micronutrients (e.g., essential fatty acids, antioxidants, vitamins, and minerals)^1^, the role of egg consumption with regard to increasing the risk for cardiovascular disease (CVD) and diabetes has been debated for decades^2–4^, mainly due to eggs’ high content of dietary cholesterol (200–250 mg per middle-sized egg)^5^.

Observational studies and randomized controlled trials (RCT) have reported conflicting data on the effect of egg consumption on lipid profiles and cardiometabolic health, with some showing beneficial and others adverse effects^6–8^. In a recent systematic review, Richard et al.^8^ reported that consuming 1–2 eggs per day for 12 weeks did not adversely affect some CVD risk factors, including plasma concentrations of high-density lipoprotein cholesterol (HDL-C), low-density lipoprotein cholesterol (LDL-C), glucose, and inflammation markers (TNF-α and IL-6) among patients with type 2 diabetes mellitus (T2DM). However, a meta-analysis of prospective studies suggested that egg consumption significantly increased the risk of CVD morbidity in patients with T2DM, while the egg-consumption level was not associated with an increased risk for CVD and cardiac mortality in the general population^7^.

To date, data on the association between dietary cholesterol intake and CVD risk were mainly reported in the United States (US) and the countries of the European Union (EU). The results of these studies might therefore not be suitable evidence for developing dietary guidelines for populations other than those of the US and the EU. There is limited information on the association between the level of egg consumption and the risk for cardiometabolic disease in the Korean population, whose average cholesterol intake is lower than that of Western populations^9,10^.

In this study, we examined the association between egg-consumption levels and CVD incidence in Korea, using the data set of a longitudinal cohort of the Ansung–Ansan region. Moreover, we tested whether the association was modified by the presence of T2DM.

## Materials and methods

### Study population

This study used data of the Ansung–Ansan cohort, obtained from the Korean Genome and Epidemiology Study. The details and design of the survey method have been described elsewhere^11^. Briefly, the Ansung–Ansan cohort study was conducted among the residents of the Ansan (urban) and Ansung (rural) areas of the Gyeonggi Province, Republic of Korea. The baseline survey was performed in 2001–2002 and involved 10030 adults aged 40–69 years; the follow-up study has been conducted every 2 years thereafter. During follow-up, data on demographics, lifestyle, health examinations and diagnostic records, metabolic indicators, and disease incidence have been collected. The overall data collection and management were handled by a trained investigator based on a standardized protocol^12^.

Among the participants of the Ansung–Ansan cohort-based survey, those with a previous diagnosis of cancer or CVD and the related diseases, and those taking drugs for these diseases were excluded from this study (*n* = 498). Participants with a total energy intake of <2092 (500 kcal) or >20920 (5000 kcal) kJ/d (*n* = 162)^13^ and those who did not have available data on egg consumption or diabetes diagnosis (*n* = 122) were also excluded. Finally, 9248 participants were included in the analysis (Supplementary Figure 1).

Informed consent was obtained from all participants included in the study, and the study was approved by the ethics committees of the Korean Centers for Disease Control and Prevention (IRB number: KU-IRB-15-EX-256-A-1) and Yeungnam University (IRB number: 7002016-E-2016-003).

### Dietary assessment

Dietary information, including that on egg consumption, was obtained from the results of the baseline (2001–2002) and second follow-up (2005–2006) surveys, using a semiquantitative food frequency questionnaire (SQFFQ) consisting of 106 food items. The details regarding the validity of the SQFFQ have been described previously^14^. Briefly, the validity of the SQFFQ was examined by comparing 12-day dietary records. The deattenuated age-adjusted, sex-adjusted, and energyintake-adjusted correlation coefficients ranged between 0.23 and 0.64. The reproducibility of the SQFFQ was acceptable, showing a mean correlation coefficient between two SQFFQs conducted with a 1-year interval of 0.45 for all nutrient intakes^14^. Each item of the SQFFQ offers nine response options for food frequency (almost never, once/month, 2–3 times/month, 1–2 times/week, 3–4 times/week, 5–6 times/week, once/day, twice/day, and thrice/day) and three response options for portion size (0.5 times the reference, the reference, and 1.5–2 times the reference).

Weighted food frequencies were calculated, considering both frequency and portion-size responses; finally, we used the mean values of the weighted food frequencies reported at the baseline, and the second follow-up to minimize the misclassification of dietary information. The total vegetable intake frequency was obtained by summating the overall servings of 15 kinds of vegetables, including Chinese cabbage, spinach, pepper leaves, lettuce, perilla leaves, balloon flower roots, carrots, crown daisies, bean sprouts, bracken, aged amber, green peppers, cucumbers, onions, and oyster mushrooms except for kimchi. Similarly, the total fruit intake frequency was calculated by determining the sum of the overall servings of 11 types of fruits, including strawberries, persimmons, mandarins, oranges, oriental melons, watermelons, pears, apples, bananas, peaches, and grapes. Furthermore, the intake frequency of red meat was determined by obtaining the overall servings of beef and pork, excluding other processed meats.

### General participant characteristics, anthropometric measurements, and biochemical variables

Demographic and lifestyle data of the participants, including age, sex, area of residence, educational level, monthly household income, smoking status, alcohol consumption, physical activity level, as well as medical and family history of diseases, were collected by trained investigators using a questionnaire. Educational level was categorized into elementary school or lower, middle school, high school, and college graduate or higher. Monthly household income was divided into <1 million Korean Republic Won (KRW), 1–<2 million KRW, 2–<4 million KRW, and ≥4 million KRW. Smoking status was determined by calculating the pack-year and converting the number of cigarettes smoked/day to the number of packs consumed. For alcohol consumption, we used the weekly intake frequency (glasses/week), and the physical activity level was determined using the metabolic equivalent of task (MET-hours/week) by calculating the time spent on exercise/week and assigning a weight based on exercise intensity^15^. The MET scores of the participants were classified as <20, 20–<40, and ≥40 MET-hours/week. Weight and height of the participants were obtained by trained technicians. The body mass index (BMI) was calculated by dividing the body weight in kilograms by the square of the height in meters. The World Health Organization’s BMI standard for Asian populations^16,17^ was used to classify the participants as underweight, normal weight, overweight, and obese. Biomarkers in blood (e.g., total cholesterol, LDL-C, HDL-C, and triglyceride) were determined by an organization using a standardized protocol.

### Assessment of cardiovascular disease incidence

Incident CVD cases were identified through biennial questionnaires, and all reported cases were confirmed by trained staff during personal interviews. CVD incidence was defined as newly diagnosed cases of myocardial infarction, coronary artery disease, cerebrovascular diseases, or based on taking medications for the aforementioned diseases or stroke.

### Statistical analysis

Baseline demographic variables, dietary factors, and health indices were compared according to the quartiles of egg consumption. Categorical variables were compared using the chi-square test. For continuous variables, means and standard errors of egg-intake quartiles were compared using the general linear-regression analysis. Multivariable-adjusted means of blood lipid concentrations were compared in association with egg-intake quartiles. Hazard ratios (HR) and 95% confidence intervals (CI) were analyzed with Cox proportional hazard regression to examine the CVD incidence by egg-intake quartiles.

Potential confounding factors were based on the results of preliminary analyses and previous literature reviews on the relevant topic. For example, the known risk factors for the main outcome and variables associated with exposure were considered. Moreover, we employed a separate model that was adjusted for variables suspected to be implicated in the causal pathway, e.g., the BMI.

The following models were performed: model 1 was unadjusted; model 2 was adjusted for age, sex, educational level, residential area, monthly household income, alcohol drinking, smoking in pack-years, and physical activity level; Model 3 was adjusted for all covariates included in model 2 plus dietary supplement use, history of hypertension and dyslipidemia, and the intake levels of total energy, vegetables, fruits, fiber, red meat, and vitamin E; and last, model 4 was adjusted for all variables included in model 3 plus BMI. We performed a collinearity test using the variance inflation factor (VIF) and condition index. None of the VIFs exceeded 2.1, and the condition indices did not exceed 3.8.

Multiple potential effect modifiers, including demographic variables, lifestyle factors, and history of medical conditions, were tested using multiplicative interaction terms in the regression model. As a result, a diagnosis of T2DM was determined as an effect modifier of the association between egg intake and CVD incidence. The *P* for trend was calculated by using the median of the egg-intake quartiles as a continuous variable. All analyses were performed using the Statistical Analysis System software (version 9.4; SAS Institute Inc., Cary, NC, USA).

## Results

The participants included in our analysis had similar characteristics as those who were excluded, including their BMI, current smoking status, physical activity level, history of dyslipidemia, and the intake levels of total vegetables, total fruits, red meat, and fiber. However, the included participants were more likely to be younger (odds ratio (OR): 0.96, 95% CI: 0.95–0.97), be men (OR: 1.22, 95% CI: 1.06–1.42), drink alcohol (OR: 1.34, 95% CI: 1.15–1.55), and have a higher educational (OR: 1.81, 95% CI: 1.39–2.37) and monthly household income levels (OR: 2.16, 95% CI: 1.51–3.09). Moreover, they were more likely to have a lower level of vitamin E intake (OR: 0.97, 95% CI: 0.95–0.99), and less likely to use dietary supplements (OR: 0.73, 95% CI: 0.60–0.87) and have a history of hypertension (OR: 0.55, 95% CI: 0.46–0.66; Supplementary Table 1).

During the average follow-up of 7.3 years, 570 incident cases of CVD were identified. The participants’ mean age at baseline was 52.0 ± 0.1 years. The average egg-intake levels were 0.1 ± 0.02, 0.7 ± 0.004, 1.6 ± 0.01, and 4.2 ± 0.04 eggs/week for the first, second, third, and fourth quartiles, respectively (Table 1). The group with the higher egg intake was younger, included more men, and showed higher educational levels than the group with the lower egg intake (*P* < 0.001 for all). Moreover, those with a higher egg intake consumed more vegetables, fruits, red meat, total energy, and dietary supplements (*P* < 0.001 for all) than their counterparts.

**Table 1:** Demographic and lifestyle characteristics of participants according to quartiles of egg consumption

	Egg-consumption levels				*P* ^a^
	Q1 (*N* = 2312)	Q2 (*N* = 2368)	Q3 (*N* = 2218)	Q4 (*N* = 2350)	
Age (years)	54.6 ± 0.2	51.6 ± 0.2	50.7 ± 0.2	51.1 ± 0.2	<0.001
Male	972 (42.0)	1178 (49.8)	1098 (49.5)	1175 (50.0)	<0.001
Body mass index (kg/m^2^)	24.6 ± 0.1	24.6 ± 0.1	24.5 ± 0.1	24.5 ± 0.1	0.1
Educational level					<0.001
≤Elementary school or lower	1066 (46.5)	778 (32.8)	602 (27.3)	596 (25.5)	
Middle school	573 (25.0)	568 (24.1)	474 (21.5)	503 (21.5)	
High school	509 (22.2)	705 (29.9)	788 (35.7)	804 (34.4)	
≥College or higher	146 (6.4)	311 (13.2)	34 (15.6)	433 (18.5)	
Residential area					<0.001
Ansung	1526 (66.0)	1172 (49.5)	846 (38.1)	997 (42.4)	
Ansan	786 (34.0)	1196 (50.5)	1372 (61.9)	1353 (57.6)	
Monthly household income (KRW)					<0.001
<1,000,000	1080 (47.6)	812 (34.8)	610 (27.9)	673 (29.0)	
1–<2,000,000	618 (27.3)	705 (30.2)	662 (30.3)	699 (30.1)	
2–<4,000,000	461 (20.3)	648 (27.7)	727 (33.2)	726 (31.3)	
≥4,000,000	109 (4.8)	171 (7.3)	189 (8.6)	225 (9.7)	
Alcohol drinking	987 (42.9)	1138 (48.3)	1098 (49.9)	1165 (49.9)	<0.001
Smoking (pack-years)	9.1 ± 0.3	9.1 ± 0.3	9.0 ± 0.3	10.1 ± 0.3	0.01
Physical activity level					<0.001
Low	1227 (55.0)	1433 (62.5)	1438 (66.5)	1435 (62.8)	
Middle	372 (16.7)	371 (16.2)	366 (16.9)	409 (17.9)	
High	631 (28.3)	488 (21.3)	358 (16.6)	439 (19.2)	
Total energy intake (kJ)	6769.7 ± 47.7	7280.6 ± 46.9	7722.4 ± 48.5	8470.1 ± 47.3	<0.001
Total vegetable intake (serving/week)^b^	20.2 ± 0.3	20.0 ± 0.3	21.5 ± 0.3	24.4 ± 0.3	<0.001
Total fruit intake (serving/week)^b^	17.3 ± 0.3	19.2 ± 0.3	18.0 ± 0.3	20.6 ± 0.3	<0.001
Egg intake (serving/week)	0.1 ± 0.02	0.7 ± 0.004	1.6 ± 0.01	4.2 ± 0.04	<0.001
Red meat intake (serving/week)^b^	1.8 ± 0.05	2.0 ± 0.04	2.2 ± 0.05	2.4 ± 0.05	<0.001
Fiber intake (g/day)^b^	6.3 ± 0.05	6.1 ± 0.05	6.1 ± 0.05	6.1 ± 0.05	0.5
Vitamin E intake (mg/day) ^b^	8.0 ± 0.1	8.1 ± 0.1	8.3 ± 0.1	8.7 ± 0.1	<0.001
Dietary supplement use	384 (16.9)	415 (17.8)	403 (18.3)	527 (22.7)	<0.001
History of disease					
Hypertension	446 (19.3)	314 (13.3)	303 (13.7)	304 (12.9)	<0.001
Dyslipidemia	62 (2.7)	59 (2.5)	52 (2.3)	50 (2.1)	0.7

Values are mean ± standard error and *n* (%)

Q quartile, KRW Korean Republic Won

^a^ *P* values are derived from χ^2^ test for categorical variables, and *P* for trends is derived from general linear regression for continuous variables

^b^ Intake levels were adjusted for total energy intake

Table 2 shows the participants’ blood lipid concentrations according to egg-intake quartiles. We did not find any significant differences in any of the indicators, including total, HDL-C, LDL-C, and triglyceride levels, in association with egg-intake quartiles.

**Table 2:** Anthropometry and blood lipid profiles according to quartiles of egg consumption

	Egg-consumption levels				*P* for trend
	Q1	Q2	Q3	Q4	
Total cholesterol (mmol/L)	4.86 ± 0.03	4.80 ± 0.03	4.81 ± 0.03	4.80 ± 0.03	0.2
HDL-cholesterol (mmol/L)	1.22 ± 0.01	1.23 ± 0.01	1.23 ± 0.01	1.23 ± 0.01	0.3
LDL-cholesterol (mmol/L)	2.88 ± 0.03	2.86 ± 0.03	2.85 ± 0.03	2.84 ± 0.03	0.3
Triglyceride (mmol/L)	1.65 ± 0.04	1.55 ± 0.04	1.60 ± 0.04	1.59 ± 0.04	0.3

Values are mean ± standard error. Adjusted for age, sex, body mass index, educational level, residential area, monthly household income, alcohol drinking, smoking in pack-years, and physical activity level

Q quartile, HDL high-density lipoprotein, LDL low-density lipoprotein

Table 3 presents the HRs and 95% CIs of CVD incidence according to egg-intake quartiles. In multivariable-adjusted models, egg-intake levels in quartiles were not associated with incident CVD.

**Table 3:** Hazard ratio (95% confidence interval) for incident CVD risk according to quartiles of egg consumption

	Egg-consumption levels				*P* for trend
	Q1	Q2	Q3	Q4	
Case	151	170	126	123	
N	2312	2368	2218	2350	
Person-years	15801	18503	15859	17197	
Model 1	1	0.95 (0.76–1.18)	0.81 (0.64–1.03)	0.74 (0.58–0.94)	0.01
Model 2	1	1.19 (0.94–1.50)	1.14 (0.89–1.46)	1.03 (0.80–1.32)	0.8
Model 3	1	1.26 (0.99–1.60)	1.22 (0.94–1.57)	1.12 (0.86–1.47)	0.8
Model 4	1	1.27 (0.99–1.61)	1.23 (0.95–1.60)	1.14 (0.87–1.49)	0.7

Model 1: unadjusted. Model 2: adjusted for age, sex, educational level, residential area, monthly household income, alcohol drinking, smoking in pack-years, and physical activity level. Model 3: additionally adjusted for dietary supplement use, history of hypertension and dyslipidemia, and the intake levels of total energy, total vegetables, total fruits, red meat, fiber, and vitamin E. Model 4: model 3 plus additionally adjusted for body mass index

CVD cardiovascular disease, Q quartile

The association between egg intake and CVD incidence was further analyzed by stratifying the participants by T2DM diagnosis (Table 4). In this analysis, we found a significant effect–modification with a 2.8-fold increase in CVD incidence in participants with T2DM and belonging to the group with the highest egg intake in comparison to those of the group with the lowest egg intake (HR: 2.81, 95% CI: 1.25–6.30); this finding also exhibited a dose–response relationship (*P* for trend: 0.02). However, no significant association between egg-intake level and CVD incidence was observed in participants without a T2DM diagnosis (HR: 1.03, 95% CI: 0.77–1.38).

**Table 4:** Hazard ratio (95% confidence interval) for CVD risk according to quartiles of egg consumption stratified by diabetes status at baseline

	Egg-consumption levels				*P* for trend
	Q1	Q2	Q3	Q4	
Diabetes					
Case	16	22	22	19	
N	184	146	138	147	
Person-years	1119	1101	877	960	
Model 1	1	1.28 (0.67–2.45)	1.67 (0.88–3.20)	1.32 (0.68–2.57)	0.5
Model 2	1	1.49 (0.73–3.03)	2.37 (1.16–4.83)	1.82 (0.89–3.72)	0.1
Model 3	1	1.60 (0.76–3.36)	3.23 (1.48–7.05)	2.43 (1.11–5.35)	0.04
Model 4	1	1.72 (0.81–3.64)	3.70 (1.65–8.30)	2.81 (1.25–6.30)	0.02
Non-diabetes					
Case	135	148	104	104	
N	2128	2222	2080	2203	
Person-years	12152	14862	12283	13670	
Model 1	1	0.92 (0.73–1.16)	0.74 (0.57–0.96)	0.69 (0.54–0.90)	0.03
Model 2	1	1.13 (0.88–1.45)	1.04 (0.79–1.36)	0.94 (0.71–1.23)	0.4
Model 3	1	1.19 (0.93–1.54)	1.08 (0.82–1.43)	1.02 (0.76–1.36)	0.8
Model 4	1	1.20 (0.93–1.55)	1.10 (0.83–1.45)	1.03 (0.77–1.38)	0.8

Model 1: unadjusted. Model 2: adjusted for age, sex, educational level, residential area, monthly household income, alcohol drinking, smoking in pack-years, and physical activity level. Model 3: additionally adjusted for dietary supplement use, history of hypertension and dyslipidemia, and the intake levels of total energy, total vegetables, total fruits, red meat, fiber, and vitamin E. Model 4: model 3 plus additionally adjusted for body mass index

CVD cardiovascular disease, Q quartile

## Discussion

This study prospectively examined the association between egg intake and CVD incidence among Korean adults by analyzing data from the Ansung–Ansan cohort. Overall, higher egg intake was not associated with an increased risk for CVD. However, after stratifying the participants by T2DM status, those with T2DM in the group with the highest egg intake had a 2.8 times higher CVD incidence than those in the group with the lowest egg intake. Conversely, we did not observe this association in participants without T2DM.

Eggs are a well-known nutritious food source that can easily supply inexpensive high-quality protein as well as micronutrients^1^. Nevertheless, egg consumption has been a main cause of health-related concerns because eggs contain a high level of dietary cholesterol and might increase the blood cholesterol level, which is in turn strongly associated with CVD^2,4^. However, prior evidence indicates that circulating cholesterols are not only derived from a diet based on animal sources but also synthesized by the liver and other organs in the human body^18,19^. Intracellular cholesterol homeostasis is precisely regulated by several physiological mechanisms involved in cholesterol biosynthesis; as such, the effect of high dietary cholesterol intake on the serum cholesterol level may be negligible^18,19^. Indeed, no measurable associations between egg-consumption levels and blood lipid profiles were observed in this study. Moreover, as eggs have high contents of antioxidants such as lutein and zeaxanthin, egg consumption might reduce the oxidation of LDL-C^20^.

Various dietary guidelines for the general population with different limitations concerning egg consumption and dietary cholesterol intake have been released^21–23^. For example, in 2015, the US Departments of Health and Human Services and Agriculture changed the dietary guideline for egg consumption from limiting egg intake to deleting the limitation altogether, but cautioning individuals to limit the intake of saturated and trans fats^22^. Several countries do not support the idea that limiting egg intake is an important dietary recommendation for the general population to maintain normal blood cholesterol levels^24^. In Korea, the 2015 Dietary Reference Intake for Koreans does not clearly indicate to limit the intake of major food sources of dietary cholesterol but does recommend to limit the dietary intake of cholesterol to <300 mg/day for the general population^23^. More specifically, the 2015 Korean guidelines for the management of dyslipidemia^25^ recommend a dietary intake of cholesterol <300 mg/day for patients with dyslipidemia or CVD to avoid excessive cholesterol intake, which is identical to European guidelines^26^.

In the present study, the association between egg-consumption quartiles and CVD incidence was modified by T2DM status. Specifically, higher egg consumption was significantly associated with an increased risk for incident CVD among participants with T2DM, whereas no evident association was detected in those without T2DM. This result is in line with several previous studies that showed a positive association between the levels of egg consumption and cardiovascular risk under metabolic conditions such as T2DM^2,7^. For example, Hu et al.^2^ investigated the association between egg-consumption levels (<1, 1, 2–4, 5–6, and 7 eggs/week) and the risk for CVD using the Health Professional Follow-Up Study (men) and Nurses’ Health Study (women) (aged 40–75 and 30–55years old, respectively). They reported that patients with T2DM who consumed ≥1 egg/day had a 2.02 times higher risk for CVD (relative risk: 2.02, 95% CI: 1.05–3.87) than those who consumed <1 egg/week. In addition, the Health, Aging, and Body Composition study conducted among elderly individuals aged 70–79 years showed that, among patients with T2DM, those with the highest egg consumption (≥3 eggs/week) had a 5.02 times higher risk for CVD (HR: 5.02, 95% CI: 1.63–15.51) than those with the lowest egg consumption (<1 egg/week)^27^.

The mechanism by which egg consumption in the diabetic state increases the risk for CVD has not been elucidated, but insulin sensitivity might affect HDL-C metabolism and cholesterol transport^3^. Thus, individuals with low insulin sensitivity, such as those with T2DM, might have elevated blood total cholesterol, very-low-density lipoprotein, and LDL-C levels when compared to the general population^28^; this cluster is associated with an increased risk for CVD. Moreover, patients with T2DM may have decreased levels of apolipoprotein E and increased levels of apolipoprotein C3, resulting in abnormal cholesterol transport^29,30^.

The dietary guidelines regarding egg and dietary cholesterol consumption for patients with T2DM vary between countries. For example, the Australian National Heart Foundation recommends a maximum intake of six eggs/week for both healthy individuals and those with T2DM^31^, whereas US guidelines have maintained a restriction on dietary cholesterol intake for diabetes patients, recommending that dietary cholesterol intake be limited to <300 mg/day (one egg contains about 200 mg of cholesterol) and <4 eggs/week^7,21,22^. The Korean Diabetes Association recommends a dietary cholesterol intake of <300 mg/day, which is identical to the dietary guideline for the general Korean population^9^. Our data support dietary cholesterol restrictions for Korean patients with T2DM but not for healthy individuals.

This study has several limitations. There might be residual confounding due to unknown or unmeasured factors in this cohort study despite the adjustments for multiple lifestyle and dietary factors that are known to be associated with CVD risk. Information on how the eggs were prepared was not collected; thus, it was not possible to evaluate the specific effect of egg consumption on the health outcome based on the cooking method. Despite these limitations, this study has several strengths, including the analysis of prospective cohort data that revealed an association between diet and disease in a prospective manner. Two repeat SQFFQs were used to reduce the error associated with the dietary survey method. Finally, to the best of our knowledge, this is the first prospective analysis on the association between egg consumption and CVD incidence conducted in the Korean population; thus, the study results are a useful scientific evidence for establishing dietary guidelines on cholesterol intake for the general Korean population and patients with T2DM who need dyslipidemia management.

In conclusion, we prospectively observed that high egg consumption might increase CVD risk in patients with T2DM in a large cohort study of Korean adults. Further investigations on the association between egg consumption and CVD risk and mortality are needed through large-scale RCTs.

## Electronic supplementary material

Supplementary table 1 Odds of being included in the final analysis

Supplementary figure 1 Flowchart depicting the inclusion process of the study participants
