# Supplementary material for: Longitudinal association between egg consumption and the risk of cardiovascular disease: interaction with type 2 diabetes mellitus
Source: Nutr Diabetes. 2018 Apr 25;8:20. doi: 10.1038/s41387-018-0033-1 (PMC5916923; doi:10.1038/s41387-018-0033-1)
Supplement: Supplementary file 1 — Supplementary table 1 Odds of being included in the final analysis [file 41387_2018_33_MOESM1_ESM.docx]

**Supplementary table 1. Odds of being included in the final analysis**

| **Variables** | **OR (95% CI) of inclusion** | ***P*** |
| --- | --- | --- |
| Age (years) | 0.96 (0.95-0.97) | <.001 |
| Male | 1.22 (1.06-1.42) | 0.01 |
| Body mass index (kg/m^2^) | 1.00 (0.97-1.02) | 0.7 |
| Educational level |  | <.001 |
| ≤Elementary school | Reference |  |
| Middle school | 1.34 (1.10-1.63) |  |
| High school | 1.48 (1.23-1.78) |  |
| ≥College | 1.81 (1.39-2.37) |  |
| Residential area |  | <.001 |
| Ansung | Reference |  |
| Ansan | 0.62 (0.53-0.72) |  |
| Monthly household income (KRW) | | <.001 |
| <1,000,000 | Reference |  |
| 1-<2,000,000 | 1.41 (1.18-1.68) |  |
| 2-<4,000,000 | 1.95 (1.60-2.39) |  |
| ≥4,000,000 | 2.16 (1.51-3.09) |  |
| Alcohol drinking | 1.34 (1.15-1.55) | <.001 |
| Current smoking | 0.89 (0.75-1.06) | 0.2 |
| Physical activity level (MET-h/week) |  | 0.3 |
| Low | Reference |  |
| Mid | 1.18 (0.95-1.48) |  |
| High | 1.04 (0.86-1.26) |  |
| Total vegetable intake (serving/week)^†^ | 1.00 (0.99-1.00) | 0.2 |
| Total fruit intake (serving/week)^†^ | 1.00 (1.00-1.01) | 0.2 |
| Red meat intake (serving/week)^†^ | 1.00 (0.97-1.03) | 0.9 |
| Fiber intake (g/day)^†^ | 1.02 (0.99-1.06) | 0.2 |
| Vitamin E intake (mg/day)^†^ | 0.97 (0.95-0.99) | 0.002 |
| Dietary supplement use (yes) | 0.73 (0.60-0.87) | <.001 |
| History of disease |  |  |
| Hypertension | 0.55 (0.46-0.66) | <.001 |
| Dyslipidemia | 0.89 (0.57-1.40) | 0.6 |
| OR, odds ratio; CI, confidence interval; KRW, Korean Republic won. †Intake levels were adjusted for total energy intake. | | |
