# Supplementary material for: Longitudinal association between egg consumption and the risk of cardiovascular disease: interaction with type 2 diabetes mellitus
Source: Nutr Diabetes. 2018 Apr 25;8:20. doi: 10.1038/s41387-018-0033-1 (PMC5916923; doi:10.1038/s41387-018-0033-1)
Supplement: Supplementary file 2 — Supplementary figure 1 Flowchart depicting the inclusion process of the study participants [file 41387_2018_33_MOESM2_ESM.pptx]

## Slide 1
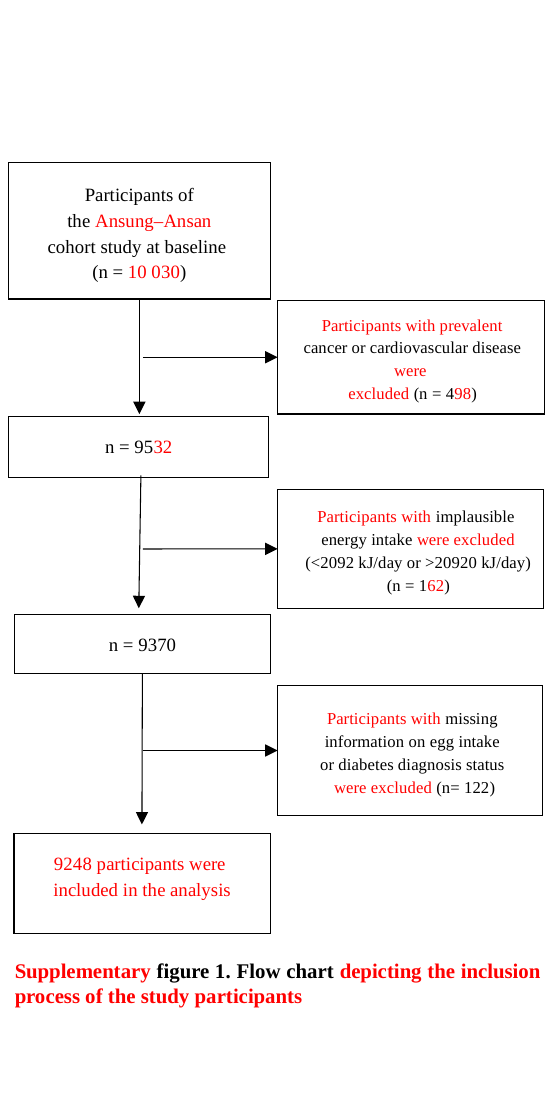

Participants of
 the Ansung–Ansan
cohort study at baseline
(n = 10 030)
Participants with prevalent cancer or cardiovascular disease were
excluded (n = 498)
n = 9532
Participants with implausible
energy intake were excluded
(<2092 kJ/day or >20920 kJ/day)
(n = 162)
n = 9370
Participants with missing
information on egg intake
or diabetes diagnosis status
were excluded (n= 122)
9248 participants were
included in the analysis
Supplementary figure 1. Flow chart depicting the inclusion process of the study participants
